# 6-Cyclo­hexyl-6,7-dihydro­dibenzo[*c*,*f*][1,5]aza­bis­mocin-12(5*H*)-yl(N→Bi) trifluoro­methane­sulfonate

**DOI:** 10.1107/S1600536811002510

**Published:** 2011-01-22

**Authors:** Nianyuan Tan, Xiaowen Zhang

**Affiliations:** a College of Chemistry and Chemical Engineering, Hunan Institute of Engineering, Xiangtan 411104, People’s Republic of China; b Key Laboratory of Pollution Control and Resource Use of Hunan Province, University of South China, Hengyang 421001, People’s Republic of China

## Abstract

In the title compound, [Bi(C_20_H_23_N)(CF_3_SO_3_)], the Bi^III^ ion shows a distorted pseudo-trigonal–bipyramidal geometry, with two C atoms and a lone electron pair of the Bi atom in equatorial positions and the N and O atoms at the apical positions. The cyclo­hexyl group is disordered over two orientations with site-occupancy factors of 0.600 (14) and 0.400 (14).

## Related literature

For the synthesis of 12-chloro-6-cyclo­hexyl-5,6,7,12-tetra­hydro­dibenzo[*c*,*f*][1,5]aza­bis­mocine, see: Zhang *et al.* (2009 ▶). For general background to the use of organobismuth compounds in catalysis, organic synthesis and medicine, see: Shimada *et al.* (2004 ▶); Kotani *et al.* (2005 ▶); Yin *et al.* (2008 ▶); Zhang *et al.* (2010 ▶). For related structures, see: Ohkata *et al.* (1989 ▶); Minoura *et al.* (1999 ▶).
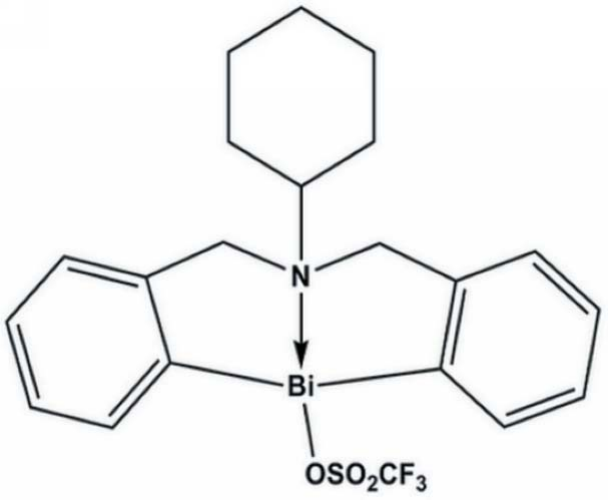


## Experimental

### 

#### Crystal data

[Bi(C_20_H_23_N)(CF_3_O_3_S)]
*M* *_r_* = 635.44
Monoclinic, 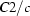

*a* = 12.6932 (13) Å
*b* = 15.0000 (14) Å
*c* = 23.037 (2) Å
β = 94.040 (2)°
*V* = 4375.2 (7) Å^3^
*Z* = 8
Mo *K*α radiation
μ = 8.20 mm^−1^
*T* = 293 K
0.31 × 0.28 × 0.11 mm

#### Data collection

Bruker SMART CCD diffractometer
Absorption correction: multi-scan (*SADABS*; Sheldrick, 1999 ▶) *T*_min_ = 0.314, *T*_max_ = 1.000
10954 measured reflections
3860 independent reflections
2920 reflections with *I* > 2σ(*I*)
*R*_int_ = 0.058

#### Refinement

*R*[*F* ^2^ > 2σ(*F* ^2^)] = 0.048
*wR*(*F* ^2^) = 0.116
*S* = 0.96
3860 reflections
305 parameters
72 restraints
H-atom parameters constrained
Δρ_max_ = 1.81 e Å^−3^
Δρ_min_ = −1.70 e Å^−3^

### 

Data collection: *SMART* (Bruker, 1997 ▶); cell refinement: *SAINT* (Bruker, 1997 ▶); data reduction: *SAINT*; program(s) used to solve structure: *SHELXS97* (Sheldrick, 2008 ▶); program(s) used to refine structure: *SHELXL97* (Sheldrick, 2008 ▶); molecular graphics: *SHELXTL* (Sheldrick, 2008 ▶); software used to prepare material for publication: *SHELXL97*.

## Supplementary Material

Crystal structure: contains datablocks I, global. DOI: 10.1107/S1600536811002510/lx2186sup1.cif

Structure factors: contains datablocks I. DOI: 10.1107/S1600536811002510/lx2186Isup2.hkl

Additional supplementary materials:  crystallographic information; 3D view; checkCIF report

## Figures and Tables

**Fig. 1.**
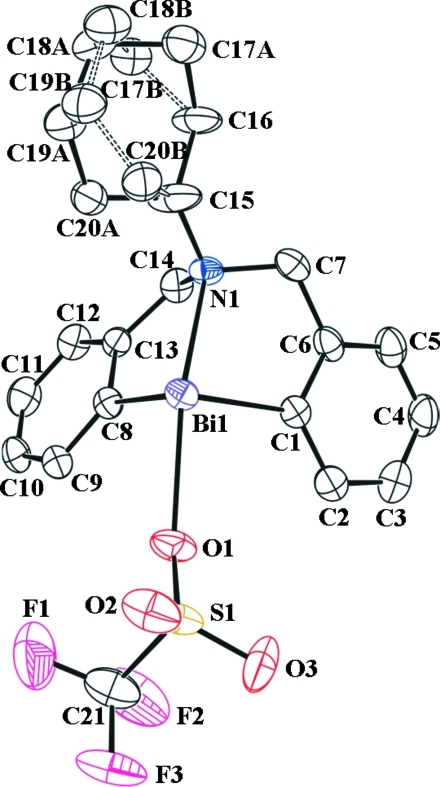
The molecular structure of the title compound with atomic numbering scheme. Displacement ellipsoids are drawn at the 30% probability level. All hydrogen atoms are omitted for clarity. The cyclohexyl group was found to be disordered over two positions and modelled with site-occupancy factors, from refinement of 0.600 (14)(Part A) and 0.400 (14)(Part B).

## supplementary crystallographic information

### Comment

The utilization of organobismuth compounds in the field of catalysis, organic synthesis and medicine has been studied intensively in recent years (Shimada *et al.*, 2004; Kotani *et al.*, 2005; Yin *et al.*, 2008; Zhang, Qiu *et al.*, 2010). The 5,6,7,12-tetrahydrodibenz[c,f][1,5]azabismocine framework is highly stable as a organobismuth Fragment because the weakly coordination exists between bismuth and nitrogen atoms on 1,5-azabismocine (Ohkata *et al.*,1989; Minoura *et al.*,1999), and therefore, is suitable for the study of organobismuth compounds bearing various groups on the bismuth atom.

In the present paper, we report the crystal structure of the title compound (Fig. 1). The central bismuth-containing part of the complex exhibits a distorted pseudo trigonal-bipyramidal structure. The C (8), C (1) atoms and a lone electron pair of the Bi atom exist at the equatorial positions while the N (1) and O (1) atoms are located at the apical positions. The Bi-C (8) and Bi-C (1) distance is 2.216 (9) Å and 2.219 (9) Å, respectively. The C (8)-Bi-C (1) angle is 96.3 (3) ° while the N (1)-Bi-O (1) angle is 151.7 (2)°(rather than 180°). The Bi-N (1) distance (2.430 (6) Å) is shorter than 2.517 (4) Å of the precursor, C_6_H_11_N(CH_2_C_6_H_4_)_2_BiCl (Zhang, Xia, Yan *et al.*, 2009). The cyclohexyl group is disordered over two positions with site-occupancy factors of 0.600 (14) (for atom labelled A) and 0.400 (14) (for atom labelled B) in Fig. 1.

### Experimental

The following procedures are recommended for synthesis of the title compound (I):12-chloro-6-cyclohexyl-5,6,7,12-tetrahydrodibenzo[c,f][1,5]azabismocine (0.522 g, 1.0 mmol) was dissolved in 15 ml THF, then a solution of AgOSO_2_CF_3 _(0.257 g, 1.0 mmol) in 10.0 ml THF was added. After the mixture was stirred in the dark at room temperature for 3 h, it was filtered. The filtrate mixed with 1.0 ml hexane was refrigerated for 24 h, giving colorless crystals (0.610 g, 96.0%).

### Refinement

All H atoms were positioned geometrically and refined using a riding model, with C-H = 0.93 Å for aryl, 0.98 Å methine and 0.97 Å for methylene H atoms, respectively. *U*_iso_(H)= 1.2*U*_eq_(C) for all H atoms. The cyclohexyl group was found to be disordered over two positions and modelled with site-occupancy factors, from refinement of 0.600 (14) (part A) and 0.400 (14) (part B), respectively. The displacement ellipsoids of disordered cyclohexyl group were restrained using command ISOR (0.01), both sets of  C atoms were restrained using the command DELU and the distances of C–C were restrained to ±1.480 (2) Å using command DFIX.

### Crystal data

**Table:**

[Bi(C_20_H_23_N)(CF_3_O_3_S)]	*F*(000) = 2448
*M**_r_* = 635.44	*D*_x_ = 1.929 Mg m^−^^3^
Monoclinic, *C*2/*c*	Mo *K*α radiation, λ = 0.71073 Å
Hall symbol: -C 2yc	Cell parameters from 3301 reflections
*a* = 12.6932 (13) Å	θ = 4.7–47.8°
*b* = 15.0000 (14) Å	µ = 8.20 mm^−^^1^
*c* = 23.037 (2) Å	*T* = 293 K
β = 94.040 (2)°	Prismatic, colorless
*V* = 4375.2 (7) Å^3^	0.31 × 0.28 × 0.11 mm
*Z* = 8	

### Data collection

**Table:**

Bruker SMART CCD diffractometer	3860 independent reflections
Radiation source: fine-focus sealed tube	2920 reflections with *I* > 2σ(*I*)
graphite	*R*_int_ = 0.058
Detector resolution: 10.00 pixels mm^-1^	θ_max_ = 25.0°, θ_min_ = 1.8°
φ and ω scans	*h* = −10→15
Absorption correction: multi-scan (*SADABS*; Sheldrick, 1999)	*k* = −15→17
*T*_min_ = 0.314, *T*_max_ = 1.000	*l* = −26→27
10954 measured reflections	

### Refinement

**Table:**

Refinement on *F*^2^	Primary atom site location: structure-invariant direct methods
Least-squares matrix: full	Secondary atom site location: difference Fourier map
*R*[*F*^2^ > 2σ(*F*^2^)] = 0.048	Hydrogen site location: difference Fourier map
*wR*(*F*^2^) = 0.116	H-atom parameters constrained
*S* = 0.96	*w* = 1/[σ^2^(*F*_o_^2^) + (0.0687*P*)^2^] where *P* = (*F*_o_^2^ + 2*F*_c_^2^)/3
3860 reflections	(Δ/σ)_max_ = 0.001
305 parameters	Δρ_max_ = 1.81 e Å^−^^3^
72 restraints	Δρ_min_ = −1.70 e Å^−^^3^

### Special details

**Table:**

Geometry. All esds (except the esd in the dihedral angle between two l.s. planes) are estimated using the full covariance matrix. The cell esds are taken into account individually in the estimation of esds in distances, angles and torsion angles; correlations between esds in cell parameters are only used when they are defined by crystal symmetry. An approximate (isotropic) treatment of cell esds is used for estimating esds involving l.s. planes.
Refinement. Refinement of F^2^ against ALL reflections. The weighted R-factor wR and goodness of fit S are based on F^2^, conventional R-factors R are based on F, with F set to zero for negative F^2^. The threshold expression of F^2^ > 2sigma(F^2^) is used only for calculating R-factors(gt) etc. and is not relevant to the choice of reflections for refinement. R-factors based on F^2^ are statistically about twice as large as those based on F, and R- factors based on ALL data will be even larger.

### Fractional atomic coordinates and isotropic or equivalent isotropic displacement parameters (Å^2^)

**Table:**

	*x*	*y*	*z*	*U*_iso_*/*U*_eq_	Occ. (<1)
Bi1	0.50647 (3)	0.51740 (2)	0.607770 (13)	0.04553 (15)	
S1	0.4571 (2)	0.75730 (17)	0.59130 (13)	0.0719 (8)	
F1	0.4536 (10)	0.8079 (8)	0.6932 (3)	0.180 (5)	
F2	0.5912 (7)	0.8544 (6)	0.6476 (5)	0.155 (4)	
F3	0.4435 (7)	0.9161 (4)	0.6299 (4)	0.133 (3)	
O1	0.5181 (6)	0.6821 (5)	0.6099 (3)	0.074 (2)	
O2	0.3476 (6)	0.7462 (5)	0.5879 (4)	0.090 (2)	
O3	0.5008 (9)	0.8007 (7)	0.5390 (3)	0.123 (3)	
N1	0.5872 (6)	0.3705 (4)	0.6105 (3)	0.0472 (17)	
C1	0.6305 (7)	0.5246 (5)	0.5447 (3)	0.042 (2)	
C2	0.6739 (8)	0.6024 (7)	0.5219 (4)	0.058 (2)	
H2	0.6487	0.6582	0.5317	0.069*	
C3	0.7538 (9)	0.5950 (8)	0.4850 (4)	0.075 (3)	
H3	0.7822	0.6464	0.4698	0.089*	
C4	0.7925 (11)	0.5138 (8)	0.4702 (5)	0.078 (3)	
H4	0.8469	0.5102	0.4453	0.093*	
C5	0.7505 (9)	0.4373 (8)	0.4923 (4)	0.069 (3)	
H5	0.7773	0.3819	0.4828	0.083*	
C6	0.6697 (8)	0.4428 (6)	0.5283 (4)	0.055 (2)	
C7	0.6192 (8)	0.3583 (6)	0.5508 (4)	0.056 (2)	
H7A	0.5578	0.3428	0.5254	0.067*	
H7B	0.6692	0.3094	0.5502	0.067*	
C8	0.6153 (7)	0.5233 (5)	0.6872 (4)	0.046 (2)	
C9	0.6218 (7)	0.5945 (6)	0.7269 (4)	0.056 (2)	
H9	0.5832	0.6464	0.7194	0.067*	
C10	0.6871 (9)	0.5859 (8)	0.7774 (4)	0.066 (3)	
H10	0.6917	0.6326	0.8040	0.079*	
C11	0.7436 (10)	0.5119 (8)	0.7886 (5)	0.075 (3)	
H11	0.7845	0.5069	0.8235	0.090*	
C12	0.7418 (8)	0.4417 (7)	0.7483 (4)	0.060 (3)	
H12	0.7842	0.3918	0.7557	0.073*	
C13	0.6770 (7)	0.4465 (6)	0.6973 (3)	0.047 (2)	
C14	0.6816 (7)	0.3762 (6)	0.6520 (3)	0.055 (2)	
H14A	0.7430	0.3870	0.6302	0.066*	
H14B	0.6915	0.3190	0.6713	0.066*	
C15	0.5142 (7)	0.2987 (5)	0.6231 (4)	0.097 (4)	
H15A	0.4587	0.3044	0.5916	0.116*	0.600 (14)
H15B	0.5114	0.3276	0.6602	0.116*	0.400 (14)
C16	0.5506 (7)	0.2034 (5)	0.6169 (4)	0.091 (4)	
H16A	0.5964	0.1872	0.6508	0.110*	0.600 (14)
H16B	0.5912	0.1985	0.5829	0.110*	0.600 (14)
H16C	0.6262	0.2053	0.6232	0.110*	0.400 (14)
H16D	0.5321	0.1815	0.5784	0.110*	0.400 (14)
C17A	0.4569 (13)	0.1385 (12)	0.6108 (7)	0.080 (6)	0.600 (14)
H17A	0.4087	0.1546	0.5779	0.096*	0.600 (14)
H17B	0.4811	0.0779	0.6056	0.096*	0.600 (14)
C18A	0.4058 (17)	0.1475 (11)	0.6656 (8)	0.078 (6)	0.600 (14)
H18A	0.4555	0.1314	0.6978	0.094*	0.600 (14)
H18B	0.3464	0.1067	0.6655	0.094*	0.600 (14)
C19A	0.3673 (15)	0.2413 (10)	0.6744 (9)	0.078 (5)	0.600 (14)
H19A	0.3341	0.2448	0.7110	0.093*	0.600 (14)
H19B	0.3145	0.2563	0.6434	0.093*	0.600 (14)
C20A	0.4565 (12)	0.3078 (10)	0.6748 (6)	0.062 (5)	0.600 (14)
H20A	0.4283	0.3677	0.6766	0.075*	0.600 (14)
H20B	0.5041	0.2983	0.7090	0.075*	0.600 (14)
C17B	0.506 (2)	0.1385 (17)	0.6610 (11)	0.075 (7)	0.400 (14)
H17C	0.5259	0.1582	0.7004	0.090*	0.400 (14)
H17D	0.5353	0.0793	0.6560	0.090*	0.400 (14)
C18B	0.371 (2)	0.136 (2)	0.6490 (13)	0.080 (8)	0.400 (14)
H18C	0.3509	0.1128	0.6105	0.096*	0.400 (14)
H18D	0.3401	0.0990	0.6777	0.096*	0.400 (14)
C19B	0.337 (2)	0.2284 (16)	0.6540 (14)	0.075 (7)	0.400 (14)
H19C	0.3388	0.2431	0.6950	0.090*	0.400 (14)
H19D	0.2643	0.2323	0.6385	0.090*	0.400 (14)
C20B	0.4007 (12)	0.3000 (16)	0.6236 (10)	0.069 (7)	0.400 (14)
H20C	0.3733	0.3011	0.5832	0.083*	0.400 (14)
H20D	0.3823	0.3568	0.6403	0.083*	0.400 (14)
C21	0.4911 (11)	0.8380 (9)	0.6433 (7)	0.094 (4)	

### Atomic displacement parameters (Å^2^)

**Table:**

	*U*^11^	*U*^22^	*U*^33^	*U*^12^	*U*^13^	*U*^23^
Bi1	0.0379 (2)	0.0445 (2)	0.0536 (2)	0.01047 (15)	−0.00109 (15)	−0.00054 (14)
S1	0.072 (2)	0.0416 (14)	0.098 (2)	0.0118 (13)	−0.0229 (16)	−0.0123 (13)
F1	0.259 (14)	0.205 (11)	0.079 (5)	0.037 (10)	0.043 (7)	−0.006 (6)
F2	0.080 (6)	0.144 (8)	0.233 (10)	−0.015 (6)	−0.033 (6)	−0.093 (7)
F3	0.135 (7)	0.045 (4)	0.216 (9)	0.011 (4)	−0.007 (6)	−0.032 (5)
O1	0.083 (6)	0.046 (4)	0.092 (5)	0.024 (4)	−0.008 (4)	−0.004 (3)
O2	0.051 (5)	0.070 (5)	0.147 (7)	0.001 (4)	0.004 (5)	−0.024 (5)
O3	0.172 (10)	0.140 (8)	0.062 (5)	0.011 (7)	0.039 (5)	0.035 (5)
N1	0.041 (5)	0.038 (4)	0.063 (4)	0.007 (3)	0.005 (3)	0.001 (3)
C1	0.034 (5)	0.050 (5)	0.041 (4)	0.010 (4)	−0.009 (4)	0.000 (4)
C2	0.055 (7)	0.062 (6)	0.055 (5)	0.009 (5)	−0.002 (5)	−0.002 (5)
C3	0.070 (8)	0.096 (9)	0.058 (6)	−0.002 (7)	0.009 (6)	0.011 (6)
C4	0.082 (9)	0.096 (9)	0.059 (6)	0.018 (7)	0.029 (6)	0.002 (6)
C5	0.071 (8)	0.082 (8)	0.054 (6)	0.027 (6)	0.008 (5)	−0.003 (5)
C6	0.062 (7)	0.058 (6)	0.044 (5)	0.020 (5)	−0.004 (4)	−0.006 (4)
C7	0.057 (6)	0.049 (6)	0.061 (5)	0.011 (5)	0.000 (5)	−0.012 (4)
C8	0.037 (5)	0.053 (5)	0.050 (5)	−0.001 (4)	0.006 (4)	−0.003 (4)
C9	0.059 (7)	0.058 (6)	0.052 (5)	0.002 (5)	0.008 (5)	−0.005 (4)
C10	0.084 (8)	0.074 (7)	0.040 (5)	−0.004 (6)	0.002 (5)	−0.014 (5)
C11	0.072 (8)	0.099 (9)	0.051 (6)	−0.005 (7)	−0.012 (6)	0.007 (6)
C12	0.047 (6)	0.074 (7)	0.059 (6)	0.015 (5)	−0.006 (5)	0.014 (5)
C13	0.043 (5)	0.047 (5)	0.049 (5)	0.009 (4)	0.008 (4)	0.006 (4)
C14	0.048 (6)	0.062 (6)	0.054 (5)	0.016 (5)	−0.005 (4)	0.007 (4)
C15	0.073 (9)	0.037 (6)	0.188 (13)	0.001 (5)	0.060 (9)	0.002 (7)
C16	0.089 (9)	0.033 (6)	0.157 (12)	−0.002 (6)	0.045 (8)	−0.009 (6)
C17A	0.092 (10)	0.063 (8)	0.084 (8)	0.001 (7)	0.000 (7)	−0.004 (7)
C18A	0.077 (10)	0.063 (7)	0.095 (9)	−0.019 (7)	0.010 (8)	0.012 (7)
C19A	0.081 (9)	0.067 (7)	0.087 (10)	−0.014 (6)	0.014 (8)	−0.001 (7)
C20A	0.064 (8)	0.057 (7)	0.066 (8)	−0.002 (6)	0.008 (7)	−0.006 (6)
C17B	0.077 (9)	0.067 (11)	0.082 (11)	−0.004 (8)	0.002 (9)	−0.001 (8)
C18B	0.080 (10)	0.069 (9)	0.089 (12)	−0.012 (8)	−0.007 (9)	0.001 (8)
C19B	0.067 (10)	0.070 (9)	0.086 (12)	−0.011 (7)	0.003 (9)	−0.002 (8)
C20B	0.075 (11)	0.063 (9)	0.069 (10)	0.007 (8)	0.006 (8)	0.006 (8)
C21	0.074 (10)	0.068 (9)	0.137 (13)	0.003 (7)	−0.015 (9)	−0.028 (8)

### Geometric parameters (Å, °)

**Table:**

Bi1—C8	2.216 (9)	C13—C14	1.487 (12)
Bi1—C1	2.219 (9)	C14—H14A	0.9700
Bi1—N1	2.430 (6)	C14—H14B	0.9700
Bi1—O1	2.475 (7)	C15—C20B	1.442 (13)
S1—O2	1.396 (8)	C15—C20A	1.447 (11)
S1—O1	1.418 (7)	C15—C16	1.5127
S1—O3	1.509 (8)	C15—H15A	0.9800
S1—C21	1.735 (12)	C15—H15B	0.9600
F1—C21	1.354 (16)	C16—C17A	1.535 (15)
F2—C21	1.290 (14)	C16—C17B	1.540 (17)
F3—C21	1.344 (14)	C16—H16A	0.9700
N1—C15	1.462 (10)	C16—H16B	0.9700
N1—C7	1.472 (10)	C16—H16C	0.9600
N1—C14	1.482 (10)	C16—H16D	0.9600
C1—C6	1.386 (12)	C17A—C18A	1.465 (13)
C1—C2	1.408 (12)	C17A—H17A	0.9700
C2—C3	1.372 (14)	C17A—H17B	0.9700
C2—H2	0.9300	C18A—C19A	1.508 (18)
C3—C4	1.366 (14)	C18A—H18A	0.9700
C3—H3	0.9300	C18A—H18B	0.9700
C4—C5	1.378 (16)	C19A—C20A	1.508 (16)
C4—H4	0.9300	C19A—H19A	0.9700
C5—C6	1.366 (13)	C19A—H19B	0.9700
C5—H5	0.9300	C20A—H15B	0.8483
C6—C7	1.528 (13)	C20A—H20A	0.9700
C7—H7A	0.9700	C20A—H20B	0.9700
C7—H7B	0.9700	C17B—C18B	1.72 (4)
C8—C9	1.403 (11)	C17B—H17C	0.9700
C8—C13	1.404 (12)	C17B—H17D	0.9700
C9—C10	1.386 (13)	C18B—C19B	1.46 (4)
C9—H9	0.9300	C18B—H18C	0.9700
C10—C11	1.337 (15)	C18B—H18D	0.9700
C10—H10	0.9300	C19B—C20B	1.54 (4)
C11—C12	1.404 (16)	C19B—H19C	0.9700
C11—H11	0.9300	C19B—H19D	0.9700
C12—C13	1.387 (12)	C20B—H20C	0.9700
C12—H12	0.9300	C20B—H20D	0.9700
			
C8—Bi1—C1	96.3 (3)	C15—C16—C17B	114.1 (12)
C8—Bi1—N1	77.2 (3)	C17A—C16—C17B	49.0 (13)
C1—Bi1—N1	75.1 (3)	C15—C16—H16A	109.3
C8—Bi1—O1	84.8 (3)	C17A—C16—H16A	109.3
C1—Bi1—O1	85.4 (3)	C17B—C16—H16A	62.4
N1—Bi1—O1	151.7 (2)	C15—C16—H16B	109.3
O2—S1—O1	116.3 (5)	C17A—C16—H16B	109.3
O2—S1—O3	115.3 (6)	C17B—C16—H16B	136.3
O1—S1—O3	111.2 (5)	H16A—C16—H16B	108.0
O2—S1—C21	108.6 (6)	C15—C16—H16C	105.4
O1—S1—C21	104.0 (5)	C17A—C16—H16C	142.4
O3—S1—C21	99.3 (7)	C17B—C16—H16C	109.0
S1—O1—Bi1	139.2 (5)	H16A—C16—H16C	49.6
C15—N1—C7	108.3 (7)	H16B—C16—H16C	62.8
C15—N1—C14	114.1 (7)	C15—C16—H16D	110.7
C7—N1—C14	110.3 (7)	C17A—C16—H16D	63.9
C15—N1—Bi1	113.6 (5)	C17B—C16—H16D	108.4
C7—N1—Bi1	103.4 (5)	H16A—C16—H16D	138.7
C14—N1—Bi1	106.5 (5)	H16B—C16—H16D	48.5
C6—C1—C2	118.3 (9)	H16C—C16—H16D	109.1
C6—C1—Bi1	114.9 (7)	C18A—C17A—C16	104.5 (13)
C2—C1—Bi1	126.8 (6)	C18A—C17A—H16D	140.6
C3—C2—C1	119.3 (9)	C18A—C17A—H17A	110.9
C3—C2—H2	120.3	C16—C17A—H17A	110.9
C1—C2—H2	120.3	H16D—C17A—H17A	83.4
C4—C3—C2	121.4 (11)	C18A—C17A—H17B	110.9
C4—C3—H3	119.3	C16—C17A—H17B	110.9
C2—C3—H3	119.3	H16D—C17A—H17B	97.6
C3—C4—C5	119.7 (11)	H17A—C17A—H17B	108.9
C3—C4—H4	120.2	C17A—C18A—C19A	111.6 (15)
C5—C4—H4	120.2	C17A—C18A—H18A	109.3
C6—C5—C4	120.0 (10)	C19A—C18A—H18A	109.3
C6—C5—H5	120.0	C17A—C18A—H18B	109.3
C4—C5—H5	120.0	C19A—C18A—H18B	109.3
C5—C6—C1	121.2 (10)	H18A—C18A—H18B	108.0
C5—C6—C7	120.5 (9)	C18A—C19A—C20A	111.5 (16)
C1—C6—C7	118.3 (8)	C18A—C19A—H19A	109.3
N1—C7—C6	111.4 (7)	C20A—C19A—H19A	109.3
N1—C7—H7A	109.4	C18A—C19A—H19B	109.3
C6—C7—H7A	109.4	C20A—C19A—H19B	109.3
N1—C7—H7B	109.4	H19A—C19A—H19B	108.0
C6—C7—H7B	109.4	C15—C20A—C19A	110.9 (12)
H7A—C7—H7B	108.0	C19A—C20A—H15B	150.0
C9—C8—C13	120.7 (8)	C15—C20A—H20A	109.5
C9—C8—Bi1	124.9 (7)	C19A—C20A—H20A	109.5
C13—C8—Bi1	114.3 (6)	H15B—C20A—H20A	90.5
C10—C9—C8	118.7 (9)	C15—C20A—H20B	109.5
C10—C9—H9	120.7	C19A—C20A—H20B	109.5
C8—C9—H9	120.7	H15B—C20A—H20B	83.8
C11—C10—C9	121.4 (9)	H20A—C20A—H20B	108.0
C11—C10—H10	119.3	C16—C17B—C18B	108.3 (19)
C9—C10—H10	119.3	C16—C17B—H17C	110.0
C10—C11—C12	120.8 (10)	C18B—C17B—H17C	110.0
C10—C11—H11	119.6	C16—C17B—H17D	110.0
C12—C11—H11	119.6	C18B—C17B—H17D	110.0
C13—C12—C11	120.1 (10)	H17C—C17B—H17D	108.4
C13—C12—H12	119.9	C19B—C18B—C17B	105 (2)
C11—C12—H12	119.9	C19B—C18B—H18C	110.7
C12—C13—C8	118.2 (8)	C17B—C18B—H18C	110.7
C12—C13—C14	120.4 (8)	C19B—C18B—H18D	110.7
C8—C13—C14	121.0 (7)	C17B—C18B—H18D	110.7
N1—C14—C13	115.1 (7)	H18C—C18B—H18D	108.8
N1—C14—H14A	108.5	C18B—C19B—C20B	117 (2)
C13—C14—H14A	108.5	C18B—C19B—H19C	108.0
N1—C14—H14B	108.5	C20B—C19B—H19C	108.0
C13—C14—H14B	108.5	C18B—C19B—H19D	108.0
H14A—C14—H14B	107.5	C20B—C19B—H19D	108.0
C20B—C15—C20A	55.1 (11)	H19C—C19B—H19D	107.2
C20B—C15—N1	129.7 (11)	C15—C20B—C19B	123.3 (16)
C20A—C15—N1	117.3 (9)	C15—C20B—H20C	106.5
C20B—C15—C16	109.0 (10)	C19B—C20B—H20C	106.5
C20A—C15—C16	110.0 (7)	C15—C20B—H20D	106.5
C20B—C15—H15A	48.5	C19B—C20B—H20D	106.5
C20A—C15—H15A	102.8	H20C—C20B—H20D	106.5
N1—C15—H15A	102.8	F2—C21—F3	106.1 (11)
C16—C15—H15A	102.8	F2—C21—F1	113.6 (13)
C20B—C15—H15B	83.5	F3—C21—F1	107.8 (12)
N1—C15—H15B	84.8	F2—C21—S1	112.5 (10)
C16—C15—H15B	122.8	F3—C21—S1	111.4 (9)
H15A—C15—H15B	123.3	F1—C21—S1	105.6 (10)
C15—C16—C17A	111.6 (8)		
			
O2—S1—O1—Bi1	28.9 (8)	C11—C12—C13—C8	0.9 (14)
O3—S1—O1—Bi1	−105.8 (7)	C11—C12—C13—C14	174.6 (10)
C21—S1—O1—Bi1	148.2 (7)	C9—C8—C13—C12	2.1 (13)
C8—Bi1—O1—S1	−153.4 (7)	Bi1—C8—C13—C12	−175.4 (7)
C1—Bi1—O1—S1	109.8 (7)	C9—C8—C13—C14	−171.7 (8)
N1—Bi1—O1—S1	156.2 (5)	Bi1—C8—C13—C14	10.9 (11)
C8—Bi1—N1—C15	111.1 (6)	C15—N1—C14—C13	−100.6 (8)
C1—Bi1—N1—C15	−148.8 (6)	C7—N1—C14—C13	137.1 (8)
O1—Bi1—N1—C15	163.0 (6)	Bi1—N1—C14—C13	25.6 (8)
C8—Bi1—N1—C7	−131.7 (6)	C12—C13—C14—N1	159.8 (8)
C1—Bi1—N1—C7	−31.6 (5)	C8—C13—C14—N1	−26.6 (12)
O1—Bi1—N1—C7	−79.8 (7)	C7—N1—C15—C20B	−101.8 (16)
C8—Bi1—N1—C14	−15.4 (5)	C14—N1—C15—C20B	134.9 (15)
C1—Bi1—N1—C14	84.7 (5)	Bi1—N1—C15—C20B	12.5 (16)
O1—Bi1—N1—C14	36.5 (8)	C7—N1—C15—C20A	−167.8 (11)
C8—Bi1—C1—C6	90.0 (6)	C14—N1—C15—C20A	69.0 (12)
N1—Bi1—C1—C6	15.1 (6)	Bi1—N1—C15—C20A	−53.4 (12)
O1—Bi1—C1—C6	174.3 (6)	C20B—C15—C16—C17A	2.1 (11)
C8—Bi1—C1—C2	−88.0 (8)	C20A—C15—C16—C17A	60.9 (10)
N1—Bi1—C1—C2	−162.9 (8)	N1—C15—C16—C17A	−160.4 (11)
O1—Bi1—C1—C2	−3.7 (7)	C20B—C15—C16—C17B	−51.3 (16)
C6—C1—C2—C3	−0.8 (13)	C20A—C15—C16—C17B	7.5 (14)
Bi1—C1—C2—C3	177.2 (7)	N1—C15—C16—C17B	146.2 (16)
C1—C2—C3—C4	−0.4 (16)	C15—C16—C17A—C18A	−62.4 (14)
C2—C3—C4—C5	0.3 (19)	C17B—C16—C17A—C18A	41.3 (17)
C3—C4—C5—C6	0.9 (18)	C16—C17A—C18A—C19A	61 (2)
C4—C5—C6—C1	−2.0 (15)	C17A—C18A—C19A—C20A	−59 (2)
C4—C5—C6—C7	177.2 (10)	C20B—C15—C20A—C19A	45.4 (14)
C2—C1—C6—C5	2.0 (13)	N1—C15—C20A—C19A	166.5 (11)
Bi1—C1—C6—C5	−176.2 (7)	C16—C15—C20A—C19A	−54.3 (14)
C2—C1—C6—C7	−177.3 (8)	C18A—C19A—C20A—C15	54 (2)
Bi1—C1—C6—C7	4.6 (10)	C15—C16—C17B—C18B	63 (2)
C15—N1—C7—C6	164.1 (7)	C17A—C16—C17B—C18B	−35.9 (16)
C14—N1—C7—C6	−70.3 (9)	C16—C17B—C18B—C19B	−56 (3)
Bi1—N1—C7—C6	43.3 (8)	C17B—C18B—C19B—C20B	44 (3)
C5—C6—C7—N1	145.0 (9)	C20A—C15—C20B—C19B	−64 (2)
C1—C6—C7—N1	−35.8 (11)	N1—C15—C20B—C19B	−162.1 (19)
C1—Bi1—C8—C9	112.7 (7)	C16—C15—C20B—C19B	38 (3)
N1—Bi1—C8—C9	−174.1 (8)	C18B—C19B—C20B—C15	−40 (4)
O1—Bi1—C8—C9	27.9 (7)	O2—S1—C21—F2	−177.8 (11)
C1—Bi1—C8—C13	−70.0 (7)	O1—S1—C21—F2	57.7 (13)
N1—Bi1—C8—C13	3.2 (6)	O3—S1—C21—F2	−57.0 (12)
O1—Bi1—C8—C13	−154.8 (6)	O2—S1—C21—F3	−58.9 (12)
C13—C8—C9—C10	−2.7 (13)	O1—S1—C21—F3	176.7 (10)
Bi1—C8—C9—C10	174.5 (7)	O3—S1—C21—F3	62.0 (12)
C8—C9—C10—C11	0.3 (15)	O2—S1—C21—F1	57.8 (11)
C9—C10—C11—C12	2.6 (18)	O1—S1—C21—F1	−66.7 (10)
C10—C11—C12—C13	−3.2 (17)	O3—S1—C21—F1	178.6 (10)
